# Clinical and prognostic analyses of 110 patients with N_3_ nasopharyngeal carcinoma

**DOI:** 10.1097/MD.0000000000013483

**Published:** 2018-12-10

**Authors:** Jing Chen, Tongxin Liu, Quanquan Sun, Fujun Hu

**Affiliations:** Department of Radiotherapy, Zhejiang Cancer Hospital, Hangzhou, Zhejiang Province, China.

**Keywords:** chemotherapy, N_3_, nasopharyngeal carcinoma, radiotherapy

## Abstract

**Objective:**

To analyze the clinical outcome and prognostic factors of N_3_ nasopharyngeal carcinomas (NPCs), provide a basis for rational treatment and improve the cure rate.

**Methods:**

A total of 110 patients with a pathologically confirmed diagnosis of N_3_ (NPC 2008 stage in China) NPC from our hospital were retrospectively included in the study conducted from April 2007 to July 2011. All patients received intensity-modulated radiation therapy. Some of these patients received various chemotherapies. The doses of the planning gross primary tumor and retropharyngeal lymph node volume, high-risk planning tumor volume, low-risk planning tumor volume, and gross tumor volume of neck lymph nodes were 6000 to 7600, 5400 to 6600, 5000 to 6000, and 6000 to 6996 cGy, respectively. The Kaplan–Meier analysis and logrank test were carried out to calculate and compare the survival rates of the patients, and the Statistical Package for the Social Sciences software version 17.0 was used for all analyses. Meanwhile, the Cox model was used to analyze the prognostic factors.

**Results:**

In this study, the 1-, 3-, and 5-year overall survival rates of the patients were 92.63%, 83.16%, and 70.53%, respectively. Based on the univariate analysis, T stage (*P* = .043) and chemotherapy (*P* *=* .003) were significant factors for survival. In the multivariate analysis, only chemotherapy influenced survival (Table 1

**Table 1:**
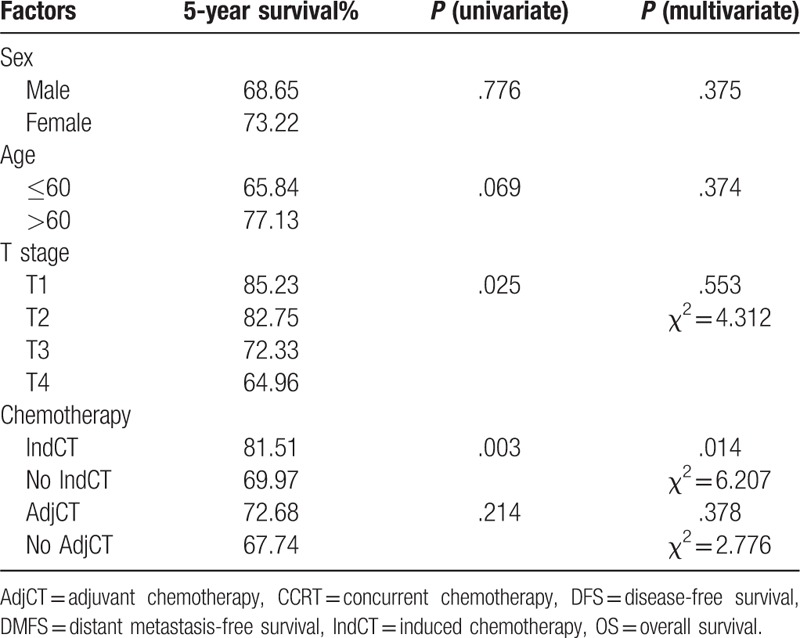
Prognostic factors affecting survival analysis of N3 nasopharyngeal carcinoma.

). Recent toxicity included radioactive oral mucosa inflammation and skin injury, and difficulty opening the mouth and hearing loss were considered late adverse reactions. None of the patients died during treatment.

**Conclusions:**

Patients with N_3_ NPC are at high risk of distant metastasis, and their 5-year survival rate is poor. The more important prognostic factors were T stage and chemotherapy. Patients with N_3_ NPC should be treated with combined chemotherapy and radiotherapy.

## 1 Introduction

Nasopharyngeal carcinoma (NPC) is a malignant tumor of the epithelial tissue, and the incidence of NPC differs in terms of age, race, and geography. The incidence rate of NPC is approximately 14.6/100,000 populations.^[1]^ The intergroup 0099 randomized trial has proven that concurrent chemoradiotherapy (CRT) followed by adjuvant chemotherapy (AdjCT) is superior to radiotherapy alone in the treatment of advanced-stage NPC.^[2]^ However, 3 earlier randomized trials have shown that adding AdjCT to radiotherapy did not improve survival.^[3–5]^

Individuals with N_3_ NPC are at high risk for distant metastasis. Local recurrence and distant metastasis are important factors that influence the survival and prognosis of patients with such condition. Radiation therapy (RT) is the primary treatment for NPC because of its inherent anatomic constraints and a high degree of radiosensitivity. However, NPC is also a chemosensitive tumor. Thus, a great deal of focus has been placed on combined RT and chemotherapy in the treatment of locoregionally advanced NPC. Thus, a retrospective analysis of 110 patients with N_3_ NPC has been conducted. Moreover, this study aimed to analyze the curative effect of the treatments and to identify the clinical features of NPC to further develop the clinical basis for the stratification of chemotherapies for locally advanced NPC.

## 2 Materials and methods

### 2.1 Clinical data

The study was approved by the ethics committee of Zhejiang Cancer Hospital. Informed consent forms were obtained from the patients. In total, 110 patients with N_3_ NPC were treated in Zhejiang Cancer Hospital from April 2007 to July 2011. Of these patients, 84 were men and 26 were women. The male-to-female ratio is 3.231:1. The age of the participants ranged from 26 to 81 years, with an average of 49.16 ± 10.61 years. The first symptoms were as follows: neck masses in 75 (68.18%) patients, nasal congestion in 12 (10.91%) patients, headache in 7 (6.36%) patients, decreased vision in 1 (0.91%) patient, neck pain in 5 (4.55%) patients, tinnitus or ear fluid flow in 30 (27.27%) patients, and epistaxis in 25 (22.73%) patients. All cases were confirmed pathologically, which include nonkeratinizing carcinoma (undifferentiated, 68 cases, 61.82%; differentiated, 39 cases, 35.45%), and low differentiated squamous cell carcinoma (3 cases, 2.73%). Metastasis in the lungs, liver, and bone was clearly identified on chest computed tomography (CT) scan or chest radiography, abdominal CT scan or B-ultrasonography, and electroconvulsive therapy before treatment. Disease stage was N_3_M_0_ (2008UICC).

### 2.2 Treatment method

#### 2.2.1 Radiotherapy

All patients received intensity-modulated RT, and the doses for planning gross primary tumor and retropharyngeal lymph node volume (PGTVnx+rn), high-risk planning tumor volume (PTV_1)_, low-risk planning tumor volume (PTV_2)_, and gross tumor volume of neck lymph nodes (GTVnd) were 6000 to 7600, 5400 to 6600, 5000 to 6000, and 6000 to 6996 cGy, respectively. The dose for important functional organs and endangered organs was limited. The maximum doses (Dmax) for the brain stem, spinal cord, optic nerve, and chiasm, temporomandibular joint, temporal lobe, crystal, mandible, and 50% volume parotid were ≤54, ≤40, ≤54, ≤50, ≤54 to 60, <8, ≤60, and ≤30 Gy, respectively. The Pinnacle 7.6 planning system was used to design the plans through synchronous integrated technology (SMART boost), and the physician outlined the target areas and normal tissues and set the prescription dose and endanger organ dose. The physical therapist established and optimized the intensity-modulated RT (IMRT) plan. The evaluation of treatment plan included the target region, endanger organ dose volume histogram, and layer evaluation of each equal section. The doctors first confirmed the treatment plan, and then, dosimetric verification was performed. Finally, RT was carried out.

#### 2.2.2 Chemotherapy

All patients received IMRT and various chemotherapy. Meanwhile, 95 patients received induced chemotherapy (IndCT), of whom, 39 and 56 received PF and TP, respectively. PF comprised nedaplatin (NDP) 75 mg/m^2^ on days 1–3, tegafur 1 g on days 1 to 3, or 5-Fu 300 to 500 mg/m^2^ via continuous intravenous injection (CIV) 72 to 120 hours for 21 days/cycle. Meanwhile, TP comprised docetaxel 75 mg/m^2^ on day 1, DDP 75 mg/m^2^ on days 1 to 3 for 21 days/cycle. Radiotherapy or concurrent chemotherapy (CCRT) was carried out after 2 to 4 cycles of IndCT. CCRT was based on NDP or DPP 80 mg/m^2^ on days 1 to 3 every 21 days. In total, 103 patients received 1 to 2 cycles of CCRT. AdjCT was performed around 1 month after radiotherapy, and a total of 53 patients received FP comprising NDP 75 mg/m^2^ on days 1 to 3, tegafur 1 g on days 1 to 3, or 5-Fu 300 to 500 mg/m^2^ via CIV 72 to 120 hours for 21 days/cycle. Seven patients received platinum-based regimens. In total, all chemotherapy regimens consisted of 2 to 3 cycles.

### 2.3 Observation and follow-up during treatment

Biochemical and routine blood tests were carried out every week during treatment. The acute responses of patients to radiotherapy and chemotherapy were recorded. Tumor regression was assessed using the nasopharyngofiberoscope or via indirect nasopharyngoscopy every 7 to 10 days. Acute toxicity and late adverse reaction evaluation criteria referred to LENT SOMA and Common Terminology Criteria for Adverse Events version 3.0 (CTCAE3.0) grade evaluation criteria. Nasopharyngofiberoscope was used and magnetic resonance imaging (MRI), chest radiography, blood test, and B-ultrasonography were conducted to assess the local control rate. Moreover, the side effects of radiotherapy were recorded. We can evaluate the control rate and identify the side effects of radiotherapy by reexamining the MRI, rhinitis fiberscopy, chest radiography, B-ultrasonography, and blood test results 1 month after the treatment to review the cases once every 3 months within 1 year, every 6 months in 3 years, and every 1 year after 5 years later. The patients should be comprehensively evaluated by assessing for thirst, neck fibrosis, and sight and hearing loss. According to the National Cancer Center of Common Toxicity Criteria version 3.0, the main adverse reactions during chemotherapy were decreased levels of granulocytes, thrombocytes, and hemoglobin. Decreased serum albumin and elevated aminotransferase levels commonly indicate liver and kidney damage. Cardiovascular abnormalities consisted of abnormal heart rhythm, electric conduction abnormalities, and cardiac insufficiency. None of the patients died during treatment.

### 2.4 Statistical analysis

The survival time was calculated from the time of diagnosis to the end of follow-up. Kaplan–Meier analysis and logrank test were carried out to calculate and compare survival rates, and the Statistical Package for the Social Sciences software version 17.0 (SPSS, China, Shanghai) was used for all analyses. Meanwhile, the Cox model was used to analyze the prognostic factors. The prognosis was evaluated in terms of gender, age, pathological type, T stage, and chemotherapy using the logrank test.

## 3 Results

### 3.1 Treatment outcome

In this study, the median survival time of the patients was 44.5 months. The 1-, 3-, and 5-year overall survival (OS) rates were 92.63%, 83.16%, and 70.53%, respectively. The univariate analysis showed that T stage (*P* = .043) and chemotherapy (*P* = .003) were significant factors of survival. Based on the multivariate analysis, only chemotherapy influenced survival rate. The median follow-up time was 70.5 months. During the follow-up, relapse was observed in 27 (24.5%) patients. Moreover, there were 8 (7.3%), 12 (10.9%), and 7 (6.4%) cases of nasopharyngeal, cervical lymph node, and nasopharyngeal and cervical lymph node recurrence, respectively. Distant metastasis occurred in 72 (65.45%) patients. Bone, lung, and liver metastases were observed in 47 (42.73%), 13 (11.82%), and 8 (7.27%) patients, respectively, and another 4 patients presented with multiple metastasis, which accounts for 3.64% of all metastatic cases. Distant metastasis was mainly observed in the first and second years after treatment. In this study, induction chemotherapy+concurrent chemoradiotherapy were compared with concurrent chemoradiotherapy alone. A significant difference was observed in terms of the 5-year OS (76.5% vs 70.3%; *P* *=* .012), DFS (71.2% vs 67.8%; *P* *=* .036), and DMFS (69.4% vs 64.9%; P = .025). Patients with N_3_ NPC should be treated with combined chemotherapy and radiotherapy.

### 3.2 Adverse reaction and evaluation

In induction chemotherapy, 3 levels of adverse reactions were observed, which were mainly leukopenia, thrombocytopenia, and cardiac insufficiency (Table 2). The adverse reactions were relieved after treatment. Two patients with grade 4 granulocytopenia and 1 patient with heart failure were delayed for chemotherapy for more than 1 week. The drug dose was decreased by 10% in the next cycle of chemotherapy if the patients obtained greater than or third-degree adverse reactions. The nasopharyngofiberoscope must be used, and nasopharynx and neck MRI and chest and abdomen CT scan should be performed to assess the disease. Regarding the RECIST standard, the changes in tumor size and duration were compared before the next treatment. The effect was classified into complete remission (CR), partial remission (PR), stable disease (SD), and progressive disease (PD). A total of 79, 14, and 2 patients presented with PR, SD, and PD, respectively. The local control rate was 97.89%.

**Table 2:**
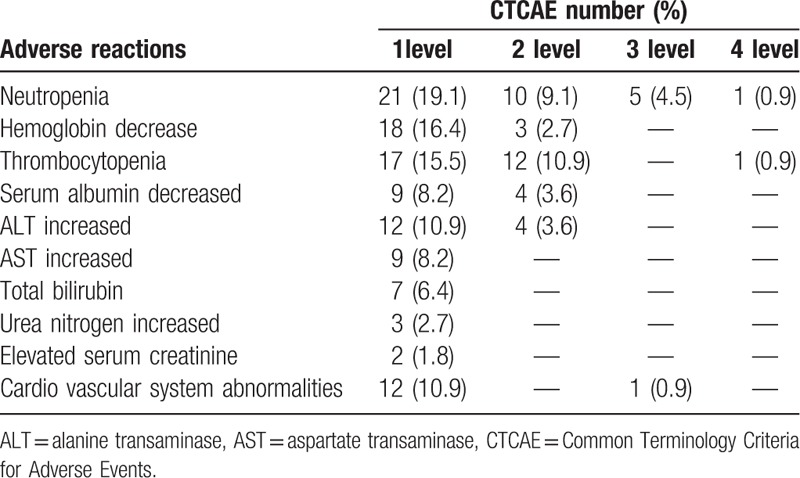
Main acute adverse events.

The treatment of patients who presented with adverse reactions during radiotherapy was not interrupted. Some patients did not undergo CCRT because they had greater than or third-degree toxic effects or adverse reactions during chemotherapy. Recent toxicity included radioactive oral mucosa inflammation and dry mouth at varying degrees. Second-degree radioactive skin injury occurred in 25 (22.73%) patients, and 32 (29.09%) patients presented with first-degree radioactive damage. These patients recovered after treatment with recombinant human epidermal growth factor gel. Late adverse reactions included difficulty in opening the mouth and hearing loss. In total, 10 (9.09%) patients presented with limited jaw movements. Auditory nerve injury was observed in 16 (14.55%) patients. Meanwhile, neck fibrosis occurred in 6 (5.45%) patients. A comprehensive review was conducted to evaluate the effect after treatments. In total, 46, 56, 9, and 4 patients presented with CR, PR, SD, and PD, respectively. The clinical beneficial rate was 96.36%. Meanwhile, the objective response rate was 88.18%.

## 4 Discussion and conclusion

Individuals with N_3_ NPC are at high risk of distant metastasis. Local recurrence and distant metastasis are important factors that influence the survival and prognosis of patients with NPC. In our study, 8 (7.3%), 12 (10.9%), and 7 (6.4%) patients presented with nasopharyngeal, cervical lymph node, and nasopharyngeal and cervical lymph node recurrence, respectively. Distant metastasis occurred in 72 (65.45%) patients. Concurrent CRT plus AdjCT has been the standard therapy for these patients for more than a decade. Moreover, the latest guidelines still include concurrent CRT plus AdjCT as an option for these patients. Significant toxicity has been observed in patients who receive AdjCT after concurrent CRT. Several multicenter trials have reported that only around 60% to 70% of patients could tolerate the entire AdjCT regimen. Therefore, many have questioned the contribution of AdjCT and advocated concurrent CRT alone.

Treatment failure of N_3_ NPC was attributed to a high rate of local recurrence and/or distant metastasis. However, advances in radiation oncology have significantly improved locoregional control, and treatment failure is now mainly due to distant metastasis. Although salvage systemic chemotherapy is usually recommended for patients with overt distant metastasis, the cure rate for metastatic NPC is extremely low.

Some retrospective studies have shown a 5-year survival rate <5%.^[6–9]^ Distant metastasis is the most important determinant of the survival rates of patients. The effect of chemotherapy is proportional to the burden of the tumor. Timely selection of N_3_ patients who need more aggressive treatment may improve the treatment outcome. RT is the primary treatment for NPC because of its inherent anatomic constraints and a high degree of radiosensitivity. However, NPC is also a chemosensitive tumor. Thus, a great deal of focus has been placed on combined RT and chemotherapy in the treatment of locoregionally advanced NPC. In patients with such condition, concurrent chemoradiotherapy was used to improve the local control rate and decrease the incidence of nasopharyngeal recurrence.^[10]^

**Figure 1:**
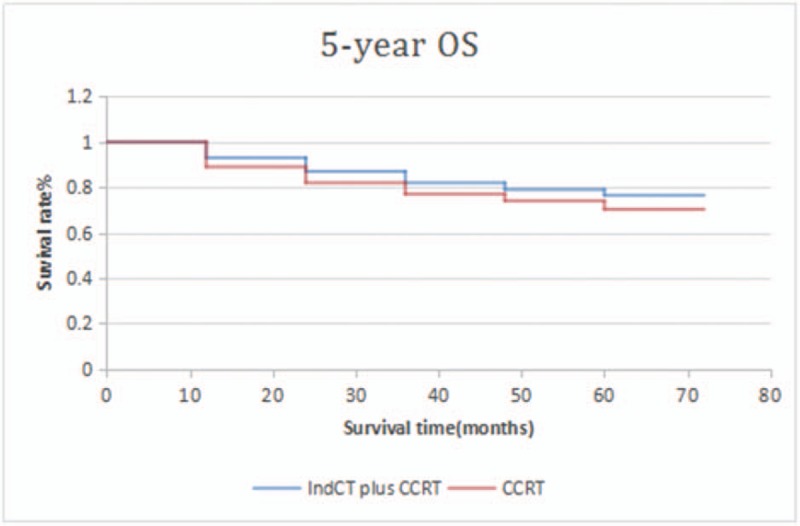
5-year OS between patients who received IndCT plus CCRT and CCRT alone. CCRT = oncurrent chemotherapy, IndCT = induced chemotherapy, OS = overall survival.

**Figure 2:**
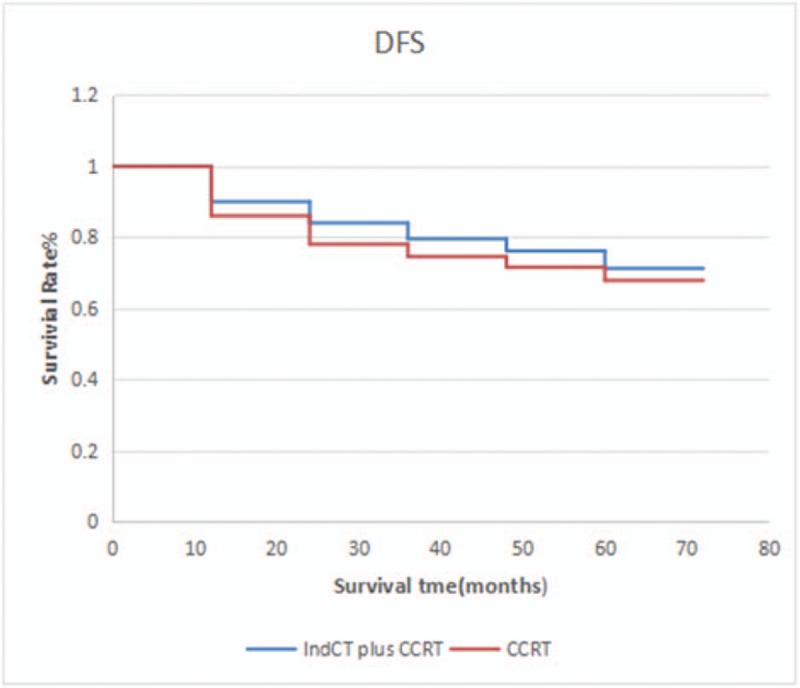
DFS between patients who received IndCT plus CCRT and CCRT alone. CCRT = oncurrent chemotherapy, DMFS = distant metastasis-free survival, IndCT = induced chemotherapy.

**Figure 3:**
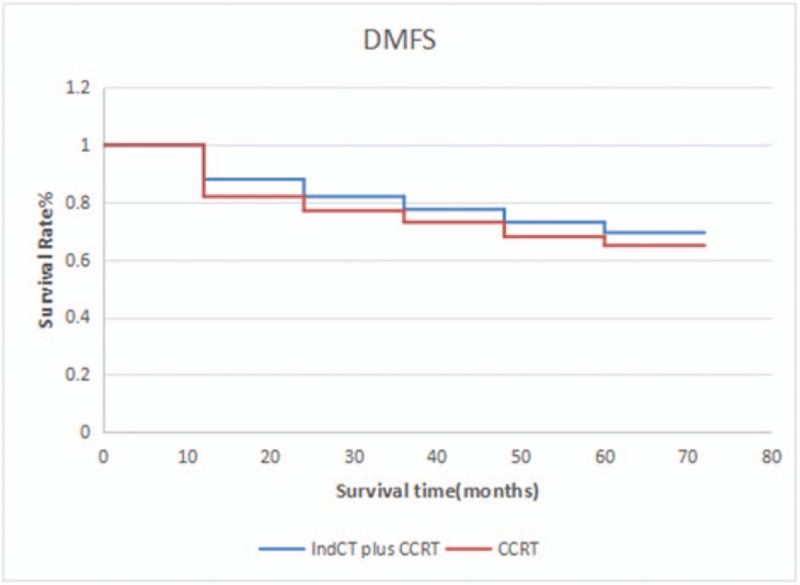
DMFS between patients who received IndCT plus CCRT and CCRT alone. CCRT = oncurrent chemotherapy, DMFS = distant metastasis-free survival, IndCT = induced chemotherapy.

In this study, induction chemotherapy+concurrent chemoradiotherapy were compared with concurrent chemoradiotherapy. A significant difference was observed in terms of the 5-year OS (76.5% vs 70.3%; *P* *=* .012), DFS (71.2% vs 67.8%; *P* *=* .036), DMFS (69.4% vs 64.9%; P = .025) (Figs. 1–3). TAN compared the patients with locally advanced NPC treated with induction chemotherapy and concurrent chemo-radiation from those receiving CCRT alone.^[11]^ Patients were stratified by N stage and randomized to induction GCP (3 cycles of gemcitabine+carboplatin + paclitaxel) followed by CCRT or CCRT alone. No significant difference was observed in terms of the 3-year OS 94 (3% vs 92.3%; *P* *=* .494, DFS (74.9% vs 67.4%; *P* *=* .362), and DMFS (83.8% vs 79.9%; *P* *=* .547). The value of induction chemotherapy in clinical settings can be influenced by the short follow-up time and non-high proportion of N_3_ patients. In the ZHANG study,^[12]^ the 4-year OS, FFS, LRFS, and DMFS of the NCT and CRT groups were 87.5% vs. 87.3% (*P* *=* .595), 78.0% vs 74.1% (*P* *=* .304), 91.2% vs 90.1% (*P* *=* .96), and 88.2% vs 84.4% (*P* *=* .154), respectively, and no statistically significant difference was observed between the 2 groups. However, a subgroup analysis has found that paclitaxel-IndCT can significantly improve the IV b (T_X_N_3_M_0_) of NPC in terms of 4-year DMFS, DFS, and OS. Some scholars believe that the screening of high-risk patients, chemotherapeutic drug selection, and dosage and follow-up time can affect the evaluation of the value of chemotherapeutic treatment. The cumulative dose of chemotherapy drugs and chemotherapy treatment were positively correlated with long-term effect.^[13]^ Multiple meta-analyses have consistently shown that combination chemotherapy reduced local recurrence risk by 27%–53%.^[14–16]^

**Figure 4:**
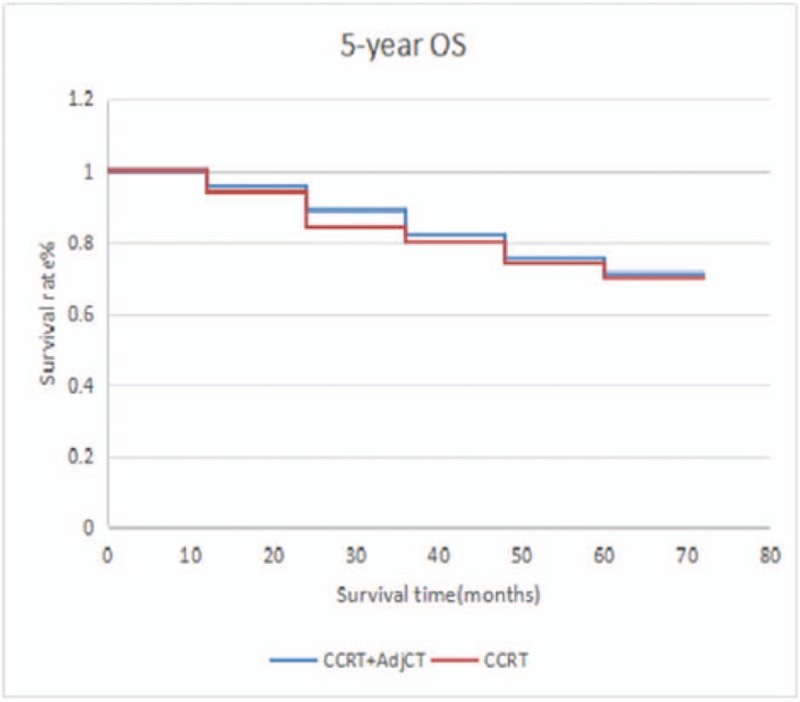
Five-year OS between patients who received CCRT plus AdjCT and CCRT alone. AdjCT = adjuvant chemotherapy, CCRT = oncurrent chemotherapy, OS = overall survival.

**Figure 5:**
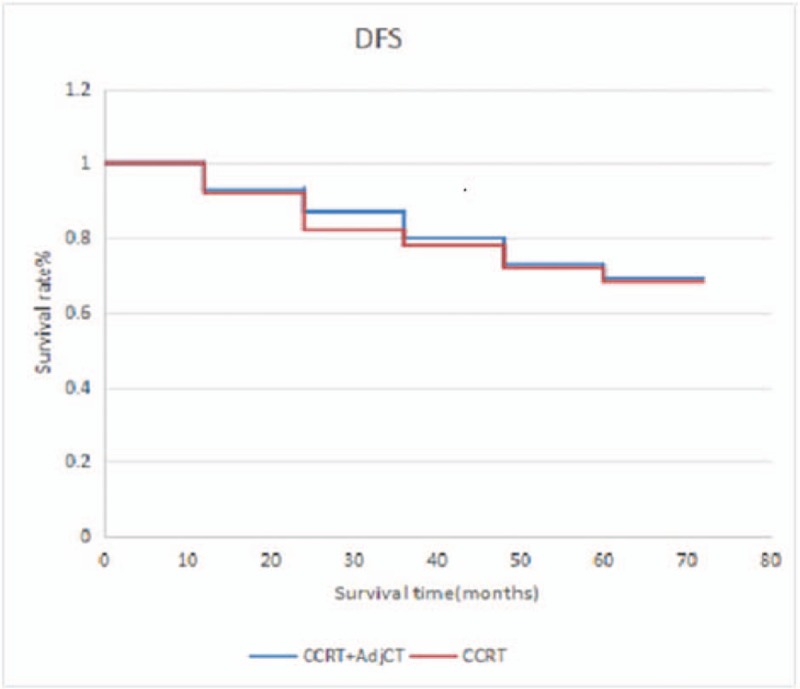
DFS between patients who received CCRT plus AdjCT and CCRT alone. AdjCT = adjuvant chemotherapy, CCRT = oncurrent chemotherapy, DFS = disease-free survival.

**Figure 6:**
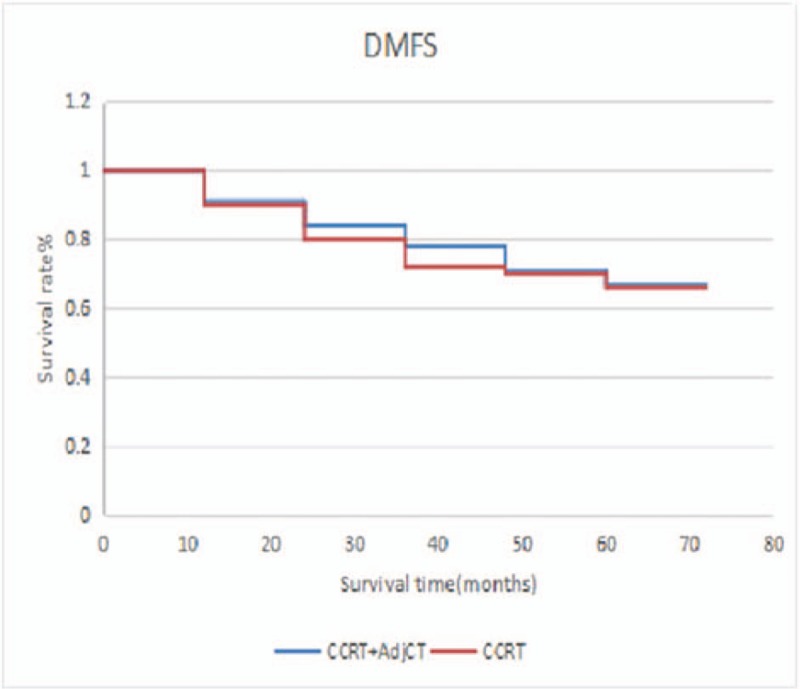
DMFS between patients who received CCRT plus AdjCT and CCRT alone. AdjCT = adjuvant chemotherapy, CCRT = oncurrent chemotherapy, DMFS = distant metastasis-free survival.

CCRT without AdjCT has also been accepted as another general treatment option with the support of phase 3 randomized trials.^[17]^ Recently, a trend toward increasing the use of IndCT has been observed, and several large phase 3 randomized trials have been conducted to compare the effect of IndCT plus CCRT versus CCRT alone.^[18]^ Despite these aggressive treatment strategies (CCRT plus AdjCT, IndCT plus CCRT, or CCRT alone), distant failure is still the most frequently reported mode of relapse.^[17,19–26]^ Because treatment only fails in some patients after initial definitive RT plus IndCT/ConCT, they believe that AdjCT is reasonable in consolidating treatment outcome for selected patients. Factors in the recurrence pattern of patients are gender, age, KPS score, and anemia.^[27]^ Whether patients with recurrence time of 1 to 2 years have unreasonable or more suggestive plans for the treatment remains to be elucidated. The recurrence of NPC after years may reflect the balance and imbalance between immune surveillance and the tumor. The recurrence in the nasopharynx can occur, and the survival rate of early local recurrence is higher than that of middle and late stage recurrence. Therefore, nasopharyngeal recurrence must be detected early. Moreover, 5 years of survival or 5 years of non-recurring survival is an artificial marker, and 5 years of survival is not a safe target for NPC. Moreover, a risk of recurrence after 5 or 10 years was observed. Therefore, follow-up time should be increased, and more active treatment for high-risk patients should be performed. In our study, 95 patients have received induction chemotherapy. In these cases, CCRT plus AdjCT was compared with CCRT, and no significant difference was observed in terms of 5-year OS (71.1% vs 70.0%; *P* *=* .24), DFS (69.9% vs 68.7%; *P* *=* .36), DMFS (67.6% vs 68.1%; P = .055) (Figs. 4–6). Some scholars believe that distant failure is still the most frequently reported mode of relapse despite the availability of aggressive treatment strategies (CCRT plus AdjCT, IndCT plus CCRT, or CCRT alone).^[26]^ In a study conducted in Italy, no differences were observed in terms of survival between the patients who received RT alone and those who received RT plus 6 monthly cycles of AdjCT, and this trial has included more patients with low-risk for distant failure and used a less active drug combination.^[5]^ Similarly, a study in Taiwan has shown the efficacy of 9 weekly cycles of adjuvant PFL (cisplatin, 5-FU, and leucovorin) and reported that the treatment was not beneficial for overall or relapse-free survivals.^[3]^ A multicenter trial in China that enrolled 508 patients did not report any significant difference in survival benefits between patients treated with CCRT and those who received CCRT plus 3 monthly cycles of adjuvant PF chemotherapy for stage III and IVB diseases.^[28]^ Adjuvant PF chemotherapy may not be extremely effective in this unselected cohort, and not all patients with locally advanced NPC present with high-risk factors for AdjCT. Moreover, another study in Taiwan has reported that AdjCT can reduce distant failure and improve OS in patients with NPC who had persistently detectable ρEBV DNA after curative RT plus induction/CCRT.^[29]^ In our study, AdjCT does not improve survival, which may be related to factors, such as the non-selection of N3 patients who are at high risk. Prospective clinical control studies with large sample sizes must be conducted in the future.

Patients with N_3_ NPC are at high risk for distant metastasis, and their 5-year survival rate is poor. The more important prognostic factors are T stage and chemotherapy. In this study, radiotherapy combined with different methods of chemotherapy has an effect on N3 NPC. Further discussion must be conducted in prospective studies.

## Author contributions

**Conceptualization:** Fujun Hu.

**Data curation:** Jing Chen, Fujun Hu.

**Formal analysis:** Jing Chen.

**Funding acquisition:** Tongxin Liu, Quanquan Sun.

**Methodology:** Jing Chen, Tongxin Liu, Fujun Hu.

**Resources:** Jing Chen, Quanquan Sun.

**Software:** Jing Chen.

**Supervision:** Fujun Hu.

**Validation:** Jing Chen.

**Visualization:** Jing Chen.

**Writing – original draft:** Jing Chen, Tongxin Liu, Quanquan Sun.

**Writing – review & editing:** Jing Chen.
